# Medico-demographic characteristics and outcomes of COVID-19 patients admitted to a provincial hospital in Center-West of Morocco

**DOI:** 10.11604/pamj.2022.42.268.35427

**Published:** 2022-08-10

**Authors:** Samira Natoubi, Rachid Amgoun, Nezha Baghdad, Mohamed Ait Elkhadir, Abdelhak Lafaf, Radouane Mouqhach, Ahmed Bouazizi, Itto Maroui

**Affiliations:** 1 Microbiology Laboratory, Provincial Hassan II Hospital, Settat, Morocco,; 2 Delegation of the Health Ministry of Settat, Casablanca-Settat Regional Health Directorate, Settat, Morocco,; 3 Department of Medicine, Unit of COVID-19, Provincial Hassan II Hospital, Settat, Morocco,; 4 Department of Basic Sciences, Research Laboratory in Oral Biology and Biotechnology, Faculty of Dental Medicine, Mohammed V University in Rabat, Rabat, Morocco

**Keywords:** Comorbidities, COVID-19, mortality, Morocco, SARS-CoV-2

## Abstract

The current Coronavirus Disease 2019 (COVID-19) pandemic that emerged in December 2019 in China continues to claim a thousand lives worldwide. This study aimed to report characteristics and in-hospital outcomes of a Moroccan COVID-19 cohort, and identify factors which predispose patients to hospitalization and mortality from COVID-19. We conducted a cross-sectional study of symptomatic COVID-19 patients referred to COVID-19 ward of the Settat Provincial Hospital, during October 2020. A confirmed COVID-19 case was defined by a positive detection of SARS-CoV-2 genome using real-time RT-PCR assay performed on nasopharyngeal swabs. Patients´ demographic and clinical characteristics were collected and analyzed using SPSS V22.0. Univariate followed by multivariate logistic regression analysis was performed to identify factors associated with mortality due to COVID-19. In total, 269 patients were reported. The median age was 64 years [IQR 54-73], 48.7% were elderly (≥ 65 years), 51.7% were men, and the case-fatality rate (CFR) was 5.58%. Males had a higher CFR (6.5%) than females (4.6%). In deceased people: 60% males, 73.3% were elderly, and oxygen saturation values of 90% or less on admission were more frequent (86.7%) than in recovered ones (10.9%). Most patients (80.3%) had at least one comorbidity: 100% of deaths, 59% older than 60 years, CFR was 6.94% and the most prevalent diseases were diabetes (54.6%), hypertension (41.7%), and cardiac disease (40.7%). The most common symptoms on presentation were dyspnea (67.7%), fever (65.4%) and cough (58.4%). Multivariable logistic regression analysis showed that only older age (AOR: 10.860, 95% CI: 3.382-34.86; p<0.001) and cardiac disease (AOR: 0.244, 95% CI: 0.074-0.799; p=0.02) were associated with higher mortality rates. Categorizing patients at admission according to risk factors identified by multivariate and also univariate analyses (mainly dyspnea) is essential to help in deciding the hospitalization priority and the strategy that will eventually reduce death risk.

## Introduction

The current coronavirus disease 2019 (COVID-19) pandemic induced by the novel beta-coronavirus Severe Acute Respiratory Syndrome Coronavirus 2 (SARS-CoV-2) that emerged in December 2019 in China, continues to claim thousand lives worldwide and firmly impact international economy and people's habits. Although Morocco was relatively spared during the first wave, COVID-19 cases number has gradually increased since lightening of containment measures from late June 2020, reaching record levels in October-November 2020. While the case-fatality rate (CFR) during this period has remained less than or equal to 1.7% [1]. Since epidemiological features and socioeconomic status vary between populations, clinical characteristics of patients and COVID-19 severity factors in Morocco may not be similar to those reported in other countries. Furthermore, defining main characteristics of COVID-19 patients and their clinical outcomes is of great importance to help in timely clinical decision-making and population health management. We thus undertook this study to characterize a cohort of COVID-19 patients admitted at the Provincial Hassan II Hospital (PHIIH) in Settat city, during October 2020. It aims to report characteristics and in-hospital outcomes of these patients and identify factors which predispose COVID-19 victims to hospitalization and mortality. Thus, this study will contribute to filling literature gaps on COVID-19 in North African hospitals.

## Methods

**Study design and setting:** we conducted a cross-sectional study of COVID-19 patients admitted at COVID-19 ward of PHIIH in Settat, Casablanca-Settat Region, Morocco; from 1^st^ to 31^st^ October 2020.

**Study population:** only laboratory-confirmed COVID-19 patients, symptomatic and requiring additional medical assistance and thus referred to the isolation ward of Settat PHIIH, during the study period, were included in this study.

**Laboratory analysis:** confirmed COVID-19 case was defined by detection of SARS-CoV-2 genome on nasopharyngeal swabs by real-time reverse transcriptase-polymerase chain reaction (RT-PCR) using GeneFinderTM COVID-19 Plus RealAmp Kit according to manufacturer's protocol (OSANG Healthcare Co, Ltd, Korea). Three genes were simultaneously amplified: envelope (E) and nucleocapsid (N) proteins, and RNA-dependent RNA polymerase (RdRp).

**Data collection:** demographics, coexisting medical disorders, clinical symptoms, peripheral oxygen saturation (SaO_2_), and RT-PCR results upon admission, were collected anonymously from patient's medical record and hospital register.

**Statistical analysis:** data analysis was performed using SPSS V-22.0 (IBM, Chicago, USA). Continuous variables were characterized by median and interquartile (IQR), whereas categorical variables were characterized by value and percentage. To compare difference between deceased and discharged patients, we used Mann-Whitney U-test and chi-squared test. To explore risk factors associated with death from COVID-19, univariable analysis was conducted for variables with p-values<0.05. Then variables with p-values<0.2 were included into multivariable logistic regression analysis. P-value<0.05 was considered statistically significant.

**Ethical considerations:** ethics approval for the study publication was provided by the Health Ministry Delegate of Settat Province (Ref. 832/2021). In fact, there is no research ethics committee in Settat Provincial Delegation, so we followed approval formalities through hierarchical procedures and obtained authorization to access the patients' data and conduct ethically this research.

## Results

In total, 269 symptomatic COVID-19 patients from Settat Province were reported. Their features together with sub-clusters of recovered and deceased are presented in Table 1. The median age was 64 years [IQR 54.0-73.0], nearly half (48.7%) were elderly (≥ 65 years), and over half (51.7%) were men. Median age was 65 years [IQR 55-73] in men and 60.5 years [IQR 53-75] in women. The CFR was 5.58%, nine deaths occurred in the intensive care unit and six in the regular ward due to lack of intensive care unit (ICU) beds. We noticed a higher CFR in males (6.5%) than females (4.6%). Among deceased, median age was 70 years [IQR 64-84], 80% were older than 60 years, and 60% were males. We observed an increase in CFR over age; it was 2.08% in patients aged 50 years or younger, rose to 6.76% in 61-70 age group, and to 8.8% in patients aged over 70 years. Lower SaO_2_ values (≤ 90%) were much more frequent among deceased patients (86.7%) than in recovered ones (10.9%). In patients with lower SaO_2_, 68% were men and 72% were 60 years or older. Various symptoms were recorded, the most prevalent were dyspnea, fever and cough. From our cohort, 53 (19.7%) patients, including a pregnant woman, had no pre-existing chronic disease. Among patients with at least one comorbidity, CFR was 6.94%, 59% were older than 60 years, 100% of deaths, and the most prevalent chronic diseases were diabetes (54.6%), hypertension (41.7%), and cardiac disease (40.7%). In deceased patients, 60% had cardiac disease and hypertension, and 33.3% had cardiac disease with diabetes and were hypertensive. Among risk factors found at initial analysis, older age [OR: 4.545, 95% CI 2.366-8.731; p<.001] ≥ 65; (OR: 9.667,95% CI 2.945-31.733 p<.001) 55-64 years], cardiac disease [(OR: 2.417, 95% CI: 1.233-4.736; p=.010)], dyspnea [(OR: 4.800, 95% CI: 2.752-8.372; p<.001)], asthenia [(OR: 2.455, 95% CI: 1.218-4.948; p=.012)], and myalgia [(OR: 2.300, 95% CI: 1.095-4.832; p=.028)] were retained at univariable analysis. However, multivariable regression analysis revealed that only older age (55-64 years (aOR: 22.80, 95% CI: 4.664-111.55; p<0.001) and ≥65 years (aOR: 10.860, 95% CI: 3.382-34.86; p<.001), and cardiac disease (aOR: 0.244, 95% CI: 0.074-0.799; p=0.02) were significantly associated with a higher risk of death from COVID-19 (Table 2).

**Table 1:** demographic and clinical characteristics of COVID-19 patients in Settat Province

Characteristics		Total (N=269)	Recovered (n=254)	Deceased (n=15)	p-value
**Gender no (%)**					0.375
	Male	139 (51.7)	130 (51.2)	9 (60)	
	Female	130 (48.3)	124 (48.8)	6 (40)	
**Age no (%)**					0.011
	25-44 years	39 (14.4)	39 (15.3)	0 (0)	
	45-54 years	30 (11.1)	29 (11.4)	1 (6.7)	
	55-64 years	69 (25.6)	66 (25.9)	3 (20)	
	≥ 65 years	131 (48.7)	120 (47.2)	11 (73.3)	
**Genes no (%)**					
	RdRp	230 (85.5)	215 (84.6)	15 (100)	0.90
	N	269 (100)	254 (100)	15 (100)	0.711
	E	202 (75.1)	189 (74.4)	13 (86.7)	0.273
**Temperature no (%)**					0.052
	< 38 °C	93 (34.6)	90 (35.4)	3 (20)	
	≥ 38 °C	176 (65.4)	164 (64.5)	12 (80)	
**Oxygen saturation, no (%)**					<0.001
	> 92%	214 (79.6)	212 (83.5)	2 (13.3)	
	≤ 92%	55 (20.4)	42 (16.5)	13 (86.6)	
**Pre-existing medical condition no (%)**					
	Diabetes	118 (43.9)	110 (43.3)	8 (53.3)	0.438
	Cardiac disease	88 (32.7)	76 (29.9)	12 (80)	<0.001
	Hypertension	90 (33.6)	81 (31.9)	9 (60)	0.170
	Chronic kidney disease	11 (4.1)	10 (3.9)	1 (6.7)	0.190
	Anemia	24 (8.9)	20 (7.9)	4 (26.7)	0.090
	thyroid disorders	7 (2.6)	7 (2.76)	0 (0)	0.517
	Chronic hepatitis	11 (4.1)	11 (4.3)	0 (0)	0.399
	Pregnancy	1 (0.4)	1 (0.4)	0 (0)	-
**Initial symptoms no (%)**					
	Cough	157 (58.4)	147 (57.9)	10 (66.7)	0.620
	Myalgia	64 (23.8)	54 (21.3)	10 (66.7)	<0.001
	Headache	25 (9.3)	22 (8.7)	3 (20)	0.194
	Anorexia	18 (6.7)	15 (5.9)	3 (20)	0.068
	Fever	176 (65.4)	164 (64.6)	12 (80)	0.260
	Diarrhea	27 (10)	23 (9.1)	4 (26.7)	0.053
	Anosmia	14 (5.2)	10 (3.9)	4 (26.7)	0.052
	Vomiting	20 (7.4)	18 (7.1)	2 (13.3)	0.509
	Dyspnea	182 (67.7)	167 (65.7)	15 (100)	0.040
	Asthenia	71 (26.4)	60 (23.6)	11 (73.3)	<0.001
	Abdominal pain	18 (6.7)	14 (5.5)	4 (26.7)	0.090

**Table 2:** odds of COVID-19-related deaths in the Settat PHIIH during October 2020

Variable		COVID-19-related death			
		Unadjusted ORs (95% CI)	P-value	Adjusted ORs (95% CI)	P-value
**Age years**					
	45-54	13.000 (1.701-99.375)	0.013	19.37 (2.00-187.24)	0.010
	55-64	9.667 (2.945-31.733)	<0.001	22.80 (4.664-111.55)	<0.001
	≥ 65	4.545 (2.366-8.731)	<0.001	10.860 (3.382-34.86)	<0.001
**Oxygen saturation %**					
	≤ 92	1.385 (0.678-2.826)	0.371		
**Pre-existing medical condition**					
	Cardiac disease	2.417 (1.233-4.736)	0.010	0.244 (0.074-0.799)	0.020
**Initial symptoms**					
	Myalgia	2.300 (1.095-4.832)	0.028	0.433 (0.132-1.419)	0.167
	Dyspnea	4.800 (2.752-8.372)	<0.001	2.273 (0.841-6.139)	0.105
	Asthenia	2.455 (1.218-4.948)	0.012	0.393 (0.118-1.308)	0.128

ORs; Odds ratios; CI ; confidence interval

## Discussion

To provide further information about COVID-19 in Morocco and identify risk factors for COVID-19 death, demographic, clinical features, and outcomes of 269 symptomatic patients monitored and treated in the COVID-19 ward of Settat PHIIH were analyzed. Found CFR remains low compared to global mortality during the study period. Only older age and cardiac disease were proven to be significantly associated with higher mortality rates. Observed CFR (5.58%) is higher than the national rate of 1.7% recorded during the study period [1]. It may be due to the fact that our study included only vulnerable patients requiring hospitalization. Nevertheless, this CFR aligns with the overall pooled mortality (5.6%) reported by Li *et al*. in a systematic review/meta-analysis using data from 86 studies and 52,808 hospitalized patients [2]. Moroccan CFR remains low compared to worldwide rate fluctuating between 2.62 and 2.90% during the same month [3]. Our results on age factor are consistent with many studies which identified older age as a risk factor associated with fatal outcomes of COVID-19 [4,5]. Increased lethality in older patients could be explained by the common multiple comorbidities occurrence with age and general lack of resilience in aging [6]. All deceased patients presented dyspnea on admission and 86.7% had decreased SaO_2_. This finding indicates that most of deaths would present impaired lung function on admission, most likely due to their not presenting earlier to the hospital. In fact, several studies reported significant association of hypoxemia and dyspnea with COVID-19 mortality [7,8]. Although dyspnea was not found to be a significant mortality predictor, specific attention is needed for patients with respiratory symptoms on admission.

Most patients (80.3%) and 100% of deaths had at least one comorbidity which could have led to their hospitalization. Of these, diabetes was the most predominant, followed by hypertension, and heart disease. Our result is in line with findings of a systematic review/meta-analysis of comorbidities in 375,859 COVID-19 cases, that reported hypertension and diabetes as the most prevalent comorbidities in COVID-19 patients [9]. Indeed, patients with hypertension and diabetes are frequently identified to have impaired immune function that could predispose them to infections [9]. Chronic hypertension, cardiac diseases and diabetes were clearly more common among deceased patients. Among deceased older than 60 years, 91.7% had cardiac disease, 83.3% were hypertensive, and 50% had diabetes. Three-quarters had cardiac disease and hypertension; 41.7% had diabetes with cardiac disease and were hypertensive. These comorbidities at an advanced age, especially in cases of late presentation or insufficient ICU capacity, could have contributed to their fatal outcome. Comorbidities are known to increase the COVID-19 death risk, indeed, Ng *et al*. study which examined comorbidities in thousand patients from 14 countries found that hypertension, diabetes and cardiovascular disease were associated with COVID-19 mortality and that the risk increased when a patient had two or more comorbidities [9]. We found a significant association between heart disease and COVID-19 mortality. Indeed, a study by Núñez-Gil *et al*. involving 2798 patients from 7 countries reported that pre-existing heart diseases represent a frailty point for COVID-19 patients and are a particular risk factor for poor prognosis [10].

**Study limitations:** first, hospitalization and in-hospital COVID-19 mortality in Settat Province during the study period were more likely underestimated since only data of patients admitted to the public PHIIH were analyzed, and other patients directly admitted to Settat's private or Casablanca hospitals were not included. Second, pre-existing medical conditions were referred on patient's self-disclosure which could lead to underestimation of the real comorbidities impact among patients because certain may be unidentified.

## Conclusion

Compared to global mortality during the study period, reported CFR remains low. Given the comparatively low number of deaths, risk factors identified through univariate analysis such as dyspnea should also be considered. Categorizing patients at admission according to identified risk factors is essential to help in deciding the hospitalization priority and the strategy that will eventually decrease death risk.

### What is known about this topic

- *Defining main characteristics of COVID-19 patients and their clinical outcomes is of great importance to help in timely clinical decision-making and population health management;*
- *The main clinico-demographic predictors of COVID-19 severity or mortality include older age, male gender, certain pre-existing medical conditions and hypoxia;*
- *The severity and mortality of COVID-19 outbreak varies between different racial/ethnic groups*.

### What this study adds

- *By presenting characteristics and outcomes of a cohort of hospitalized COVID-19 patients in Center-West of Morocco, this study attempts to partially contribute to filling gaps in the literature about COVID-19 in North African hospitals;*
- *Among characteristics assessed, only older age and heart diseases have been significantly associated with a higher risk of death from COVID-19;*
- *Any Moroccan COVID-19 patient showing one of these features or other identified risk factors from univariate analysis such as dyspnea should be immediately hospitalized and given special attention during treatment to reduce the death risk*.
